# Psychiatry in times of crisis -quo vadis? Mental health and war: the role of Lancet Psychiatry Commission on Mental Health in Ukraine

**DOI:** 10.1192/j.eurpsy.2025.32

**Published:** 2025-08-26

**Authors:** I. Pinchuk

**Affiliations:** Institute of Psychiatry, the Taras Shevchenko National University of Kyiv, Kyiv, Ukraine

## Abstract

**Abstract:**

With both the Russian invasion of Eastern Ukraine and Crimea in 2014, and the war that began in February 2022, Ukraine has faced extensive damage, numerous casualties, widespread displacement of people, and significant psychological trauma. These events have strained health care and, particularly, mental health care systems that were ill-equipped to handle such stresses.

The Commission was created in February 2023, coincident with the first anniversary of the Russian attacks. Prof. Irina Pinchuk was invited to lead The *Lancet Psychiatry* Commission on Mental Health in Ukraine, with Prof. Norbert Skokauskas and Prof. Bennett Leventhal as Co-chairs. The Commission brought together 40 experts in mental health, economics, law, and science from 12 countries, formed into five workgroups (Clinical Services, Clinical Training, Research, Forensics and Legal Advocacy, and Finance).

The Commission recommends a stepped-care model in which low-intensity services are provided by non-specialist personnel, with proper training, integrated in primary care, augmented by digital tools, all directed to moving individuals from institutional to community-based services; Developing a multidisciplinary mental health research infrastructure that is adequately funded and initially focused on relevant issues such as PTSD, the war’s impact on Ukrainians, and innovative service models for Ukraine; Policy and legislative changes; Increase in Ukraine’s mental health spending, to 4.5% of total healthcare expenditures.

The Lancet Commission supports Ukraine’s goals to end the war, provide evidence-based services for its citizens and to join the global community with improved resources clinical education, research, legal structures, funding, and care.

**Disclosure of Interest:**

None Declared

